# A pre-operatively diagnosed advanced abdominal pregnancy with a surviving neonate: a case report

**DOI:** 10.1186/s13256-015-0712-7

**Published:** 2015-10-08

**Authors:** Wondimu Gudu, Delayehu Bekele

**Affiliations:** 1 Department of Obstetrics & Gynecology, Karamara Regional Referral Hospital, P.O. Box 238, Jigjiga, Somali Regional State Ethiopia; 2 Department of Obstetrics & Gynecology, Saint Paul’s Hospital Millennium Medical College, P.O. Box 143079, Addis Ababa, Ethiopia

**Keywords:** Advanced abdominal pregnancy, Alive neonate, Ectopic pregnancy

## Abstract

**Introduction:**

Abdominal pregnancy is a rare form of ectopic pregnancy with a high rate of maternal and fetal complications. Most of the reported cases are diagnosed in early trimesters, usually after presenting with complications. Advanced abdominal pregnancy poses a huge diagnostic and management challenge, particularly in low-income countries. A term abdominal pregnancy diagnosed pre-operatively and resulting in a surviving neonate is quite rare.

**Case presentation:**

A 35-year-old, para 2, unbooked, Ethiopian Somali woman presented with amenorrhea of 9 months’ duration, abdominal pain, and painful fetal movements of 4 months’ duration. Her physical examination revealed a uterus sized 36 weeks’ gestation with easily palpable fetal parts. Her laboratory test results were unremarkable except for mild anemia. Her ultrasound findings were suggestive of abdominal pregnancy. Laparotomy was done to salvage an alive healthy neonate from the peritoneum with removal of placenta implanted on the right broad ligament. The mother had a smooth post-operative course.

**Conclusions:**

An advanced abdominal pregnancy diagnosed pre-operatively with delivery of a surviving neonate is rare. A high index of suspicion and thorough clinical and ultrasound evaluation are crucial to making an early diagnosis. This is particularly important in areas where advanced imaging technologies are not readily available. Timely surgical intervention is imperative to avert maternal and fetal complications.

**Electronic supplementary material:**

The online version of this article (doi:10.1186/s13256-015-0712-7) contains supplementary material, which is available to authorized users.

## Introduction

Advanced abdominal pregnancy (AAP) is defined as a pregnancy of over 20 weeks’ gestation with a fetus living, or showing signs of having once lived and developed, in the mother’s abdominal cavity [1, 2]. Abdominal pregnancy has an incidence of about 1 in 400 to 1 in 50,000 deliveries, and the variable incidence depends on the characteristics of a particular geographic region [1]. It is associated with a high rate of maternal and fetal complications.

The clinical presentation is variable, and the optimal approach to the evaluation and management of abdominal pregnancy is not well determined [2, 3]. A high index of suspicion and thorough clinical and ultrasound examinations are crucial to diagnosing abdominal pregnancy. Computed tomography (CT) and magnetic resonance imaging (MRI) may help in making the diagnosis and planning intra-operative care [4]. Timely surgical intervention is imperative to preventing maternal and fetal catastrophe. In this report, we describe a rare case of advanced secondary abdominal pregnancy diagnosed pre-operatively and salvaged by laparotomy with excellent maternal and neonatal outcomes.

## Case presentation

A 35-year-old, married, para 2 (both stillbirths), Ethiopian Somali woman presented to Karamara Regional Hospital on 16 August 2014 with diffuse abdominal pain and recurrent vaginal spotting of 4 months’ duration associated with nausea, anorexia, and occasional vomiting. She had been amenorrheic for 9 months and had noticed painful fetal movements during the previous 2 months. She had never attended antenatal care. Both her previous deliveries had been spontaneous vaginal deliveries that ended in stillbirths.

Her physical examination revealed stable vital signs, mild pallor, distended abdomen with a uterus sized at 36 weeks’ gestation, easily palpable fetal parts, fetus in transverse position, and diffuse abdominal tenderness but no guarding or rigidity. There was no sign of fluid in her peritoneum. The fetal heart beat was positive. The cervix was firm, smooth, and closed.

**Fig. 1:**
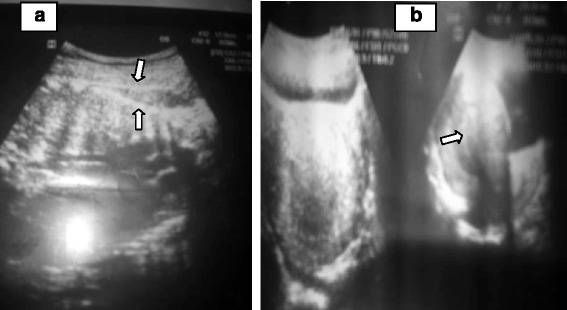
Ultrasound images of abdominal pregnancy. **a** The fetal spine (*lower arrow*) can be seen just under abdominal wall echo (*upper arrow*) without intervening myometrial tissue. **b** An empty uterus

The laboratory findings were all unremarkable, except for mild anemia (hemoglobin of 10g/dl). Ultrasound scanning revealed an enlarged, otherwise empty uterus with a thick endometrial echo and a 37 weeks’ gestation singleton, viable pregnancy in transverse lie with no intervening myometrial tissue (echo) between fetal parts and the abdominal wall (Fig. 1). There was an ill-defined, echo-complex mass in the right adnexa measuring 12cm×10cm with high Doppler flow. No amniotic fluid pool could be seen, but there was a small amount of free peritoneal fluid in the left paracolic gutter. The sonographic diagnosis was viable third-trimester abdominal pregnancy.

**Fig. 2:**
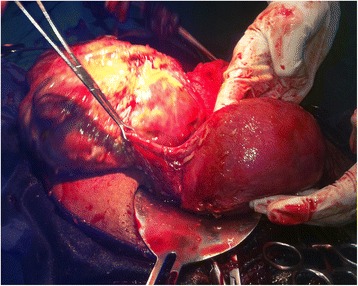
Photograph showing the implantation of the placenta on the right broad ligament

**Fig. 3:**
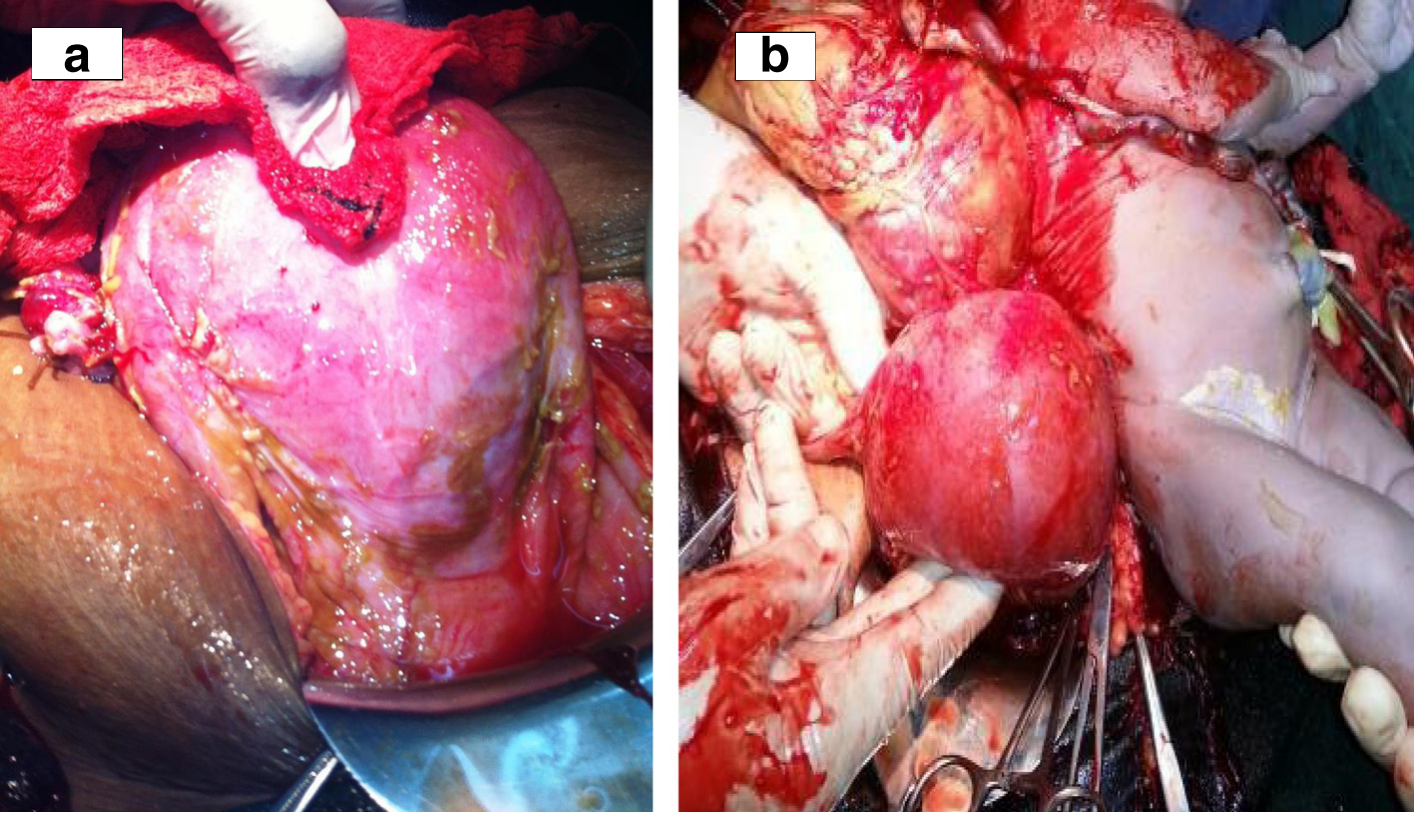
Photographs showing intact uterus (**a**) and the uterus, placenta, and the neonate after laparotomy (**b**)

The woman was counseled on the diagnosis, and informed consent was obtained for surgery. After securing 4 U of whole blood, laparotomy was done through a subumbilical midline incision that revealed the following findings: an alive, meconium-stained male neonate retrieved free from the peritoneum, weighing 2600g, and having Apgar scores of 7 and 9 in the first and fifth minutes, respectively. The baby had no gross congenital anomalies. The movie file in Additional file 1 shows the surgical procedure in detail. Fine adhesions involving the placental membranes, the omentum, and part of ileum obscured access to the pelvic cavity. Adhesiolysis revealed a fragile placenta localized in the right adnexa with a network of engorged vascular communications with the right ovarian, fallopian tube, and broad ligament vessels. The fimbrial end of the right tube was distorted and embedded in the base of the placenta (Fig. 2). The uterus was enlarged but otherwise normal-looking and intact (Fig. 3). Both ovaries and the left fallopian tube were normal. Right salpingectomy and ligation and transfixion of the vessels at the base of the placenta were done to remove the placenta completely without causing significant bleeding. An abdominal drain was left in situ. The patient was transfused with 2 U of whole blood. Both the woman and the neonate had smooth post-operative courses and were discharged on the fifth post-operative day.

## Discussion

Ectopic pregnancy is estimated to occur in 1–2% of pregnancies [3]. Over 90% are located in the fallopian tube, and the remainder implant in locations such as the abdomen, cesarean (hysterotomy) scar, cervix, and ovary [3]. Abdominal pregnancy accounts for 1.4% of ectopic pregnancies [2, 3]. Most cases of abdominal pregnancies are secondary to aborted or ruptured tubal pregnancy [2]. This was evident in our patient as the pregnancy was implanted in the right broad ligament, which most likely resulted from tubal abortion because the right tube was distorted and embedded in the placental bed.

The clinical presentation of abdominal pregnancy is variable, based on the location and the gestational age at diagnosis [3]. Similarly to other previous reports of AAPs, our patient presented with suspicious symptoms and/or signs, including painful fetal movement, abdominal pain, easily palpable fetal parts, and transverse lie [2, 5–7]. She had also recurrent vaginal bleeding that could be ascribed to endometrial response to high estrogen levels in pregnancy [3]. Very rarely reported presentations are acute abdomen and shock due to severe intra-abdominal hemorrhage and as a case of failed induction [2, 3].

A high index of suspicion is vital in making the diagnosis of AAP. The classic ultrasound finding of absence of myometrial tissue between maternal bladder and fetal parts was evident in our patient, along with an empty uterus and other suggestive findings, including poor definition of the placenta, oligohydramnios, and transverse lie [3, 8]. An AAP may be missed even after repeated ultrasound scanning if a thorough evaluation is not done, which was evident in many previous case reports. CT and MRI are useful for confirming the diagnosis, distinguishing anatomic relationships and potential vascular connections, and assessing placental adherence [4]. Our patient presented late, neglecting all the unusual symptoms in pregnancy, and she lives in an area where advanced imaging technologies are lacking. It is to be noted, however, that, unlike most previous reports of AAPs diagnosed intra-operatively, in our patient the diagnosis was made pre-operatively upon her initial presentation.

It is widely accepted that the treatment of AAP is immediate surgery once fetal viability is achieved. But the management of the placenta is controversial. In our patient, laparotomy was done immediately upon the diagnosis of AAP, and the fetus was delivered easily. Fortunately, the placenta was also retrieved relatively easily without significant bleeding, as the vessels supplying the placental bed were easily ligated and there were no major adhesions. An alternative management of the placenta suggested by most authors is ligating the umbilical cord and leaving the placenta in situ with or without administration of methotrexate. This is preferred, especially if the placenta is implanted on vital abdominal organs or major vessels [2, 8, 9]. More recently, expectant management of AAP to gain fetal maturity has been reported with some success in a few cases [10].

Maternal and fetal prognoses are generally grave in AAP. Maternal death in these cases is usually the result of uncontrollable hemorrhage and has been reported in as many as 20% of cases [3]. Perinatal mortality is also high (40–95%), and congenital malformations and deformations are reported in 21.4% of fetuses [11]. In our patient, both maternal and fetal outcomes were excellent. The mother did not have any significant intra-operative bleeding, as the removal of the placenta was technically easy. Despite the lack of surrounding amniotic sac and fluid, the neonate did not have any of the reported anomalies and/or deformities. The fetus appeared that it could survive until term because of a rich placental vascular supply from tubo-ovarian and broad ligament vessels. Importantly, the early diagnosis and timely management of the case were crucial in ensuring successful maternal and fetal outcomes.

## Conclusions

An AAP diagnosed pre-operatively and resulting in the delivery of a surviving neonate is rare. A high index of suspicion, a thorough clinical evaluation, and comprehensive ultrasound scanning are crucial to making an early diagnosis, particularly in areas where advanced imaging technologies are not readily available. Timely surgical intervention is imperative to avert maternal and fetal complications.

## Consent

Written informed consent was obtained from the patient for publication of this case report and any accompanying images. A copy of the written consent is available for review by the Editor - in - Chief of this journal.

## Additional file

Additional file 1 **Laparotomy for delivery of the baby.** An alive neonate delivered free from peritoneum after opening of the abdomen. (MP4 19227 kb)

## Abbreviations

AAP: advanced abdominal pregnancy
CT: computed tomography
MRI: magnetic resonance imaging
